# Voluntary Generation of Hyperchaotic Visuo-Motor Patterns

**DOI:** 10.1038/s41598-019-50369-9

**Published:** 2019-09-25

**Authors:** Hugh R. Wilson

**Affiliations:** Centre for Vision Research, York University, Toronto, Canada

**Keywords:** Cognitive neuroscience, Applied mathematics

## Abstract

Unpredictable escape behaviour is an integral part of the repertoire of many prey species. Many social species, including humans, also employ unpredictability to enhance their chances of success in social interactions with conspecifics. However, it is unclear what mechanisms provide the behavioural and neural bases of this unpredictability, which might result either from randomness or from chaotic dynamics. A novel paradigm described here demonstrates that unpredictable behaviour generated voluntarily in a simple visuomotor task represents high dimensional chaos, or hyperchaos, but not randomness. The exponential decay of predictability was also shown to be longer in older adults. As chaos has been observed among cortical neurons, this provides a plausible neural basis for hyperchaos. These results thus provide evidence that voluntary unpredictable behaviour can result from neural hyperchaos. This work has implications for the study of autism, aftereffects of concussions, early dementia, and the concept of free will.

## Introduction

There has been an ongoing controversy concerning whether humans can generate random behavioural sequences^1^. The literature, as summarized by Wagenaar in 1972^2^, concluded that randomness was not within the human repertoire. Treisman and Faulkner^3^ challenged this by postulating a random process in the brain that was rendered non-random through slow decay of a signal detection criterion. Finally, Neuringer and Voss^4^ attempted to show that humans could learn the chaotic behaviour of the logistic mapping, but this has been sharply criticized as learning of only the parabolic constraint on the mapping rather than the logistic mapping per se^5^. All of these earlier studies may be faulted for forcing humans into using an unnaturally small number of categories (median 8)^6^ or into adopting an unnatural, one-dimensional mapping^7^. In the work reported here, an effective continuum of “categories” (>5.0 * 10^5^) was used in two-dimensions, and participants were asked to produce random or unpredictable movement sequences.

In recent years, the concept of Machiavellian intelligence has been proposed as a major aspect of human social interactions and decisions^8^. The key concept is that unpredictable behaviour is sometimes the most effective way of controlling others. Furthermore, unpredictability of movement direction is of obvious value in avoiding predators^9^, and is mimicked by humans deceiving defenders in football or basketball. Clearly, therefore, humans and many other animals appear to generate behaviour that is sufficiently unpredictable to fool others in many situations. But what is the nature of this unpredictability? Given the history outlined above, the suggestion pursued here is that deterministic chaos represents the key.

In this paper, participants were requested to generate a sequence of randomly or unpredictably located clicks within a circular region of a computer monitor. The data were then evaluated using a test for randomness plus three tests of chaos. The results for all participants indicated that these voluntarily generated click sequences were not random but rather the result of high order chaos, or “hyperchaos”^10,11^. (Hyperchaos refers to the condition where two or more Lyapunov exponents of a dynamical system are positive^10,11^.) Furthermore, unpredictability ensued more slowly in the older population than in the young, although both groups still exhibited hyperchaos. Appropriate neural models^12,13^ are capable of producing comparable levels of hyperchaos, thus demonstrating that cortical networks could in fact generate such behaviour.

## Methods

**Figure 1:**
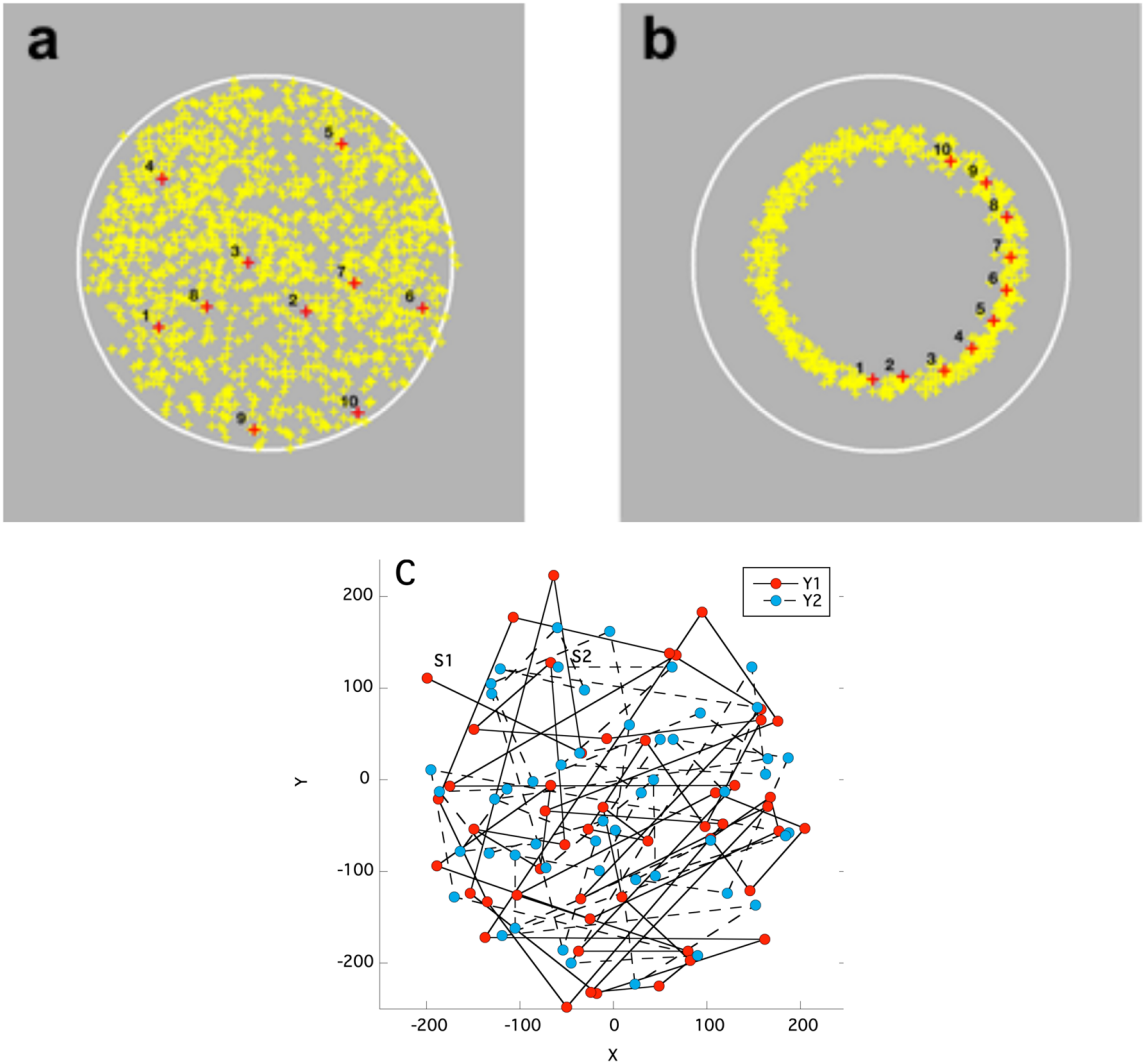
Location of all experimental click positions for one typical observer. (**a**) Click locations (yellow crosses) generated when requested to perform as randomly or unpredictably as possible. (**b)** Locations when requested to click around an imagined, invisible circle of approximately half the diameter of the visible white outline. In each panel a sequence of ten clicks from the middle of the experiment are numbered in order and shown as red crosses to give a flavour for the temporal variation of successive click locations. In the actual experiments, only the white background circle on a grey background plus the current location of the cursor cross-hairs were visible. (**c**) The first 50 clicks in the experiment (red) and its repeat (blue). S1 and S2 indicate the first click in the first and second sequence respectively. There is no statistically significant correlation between the sequences.

Participants sat in a dimly illuminated room facing a computer monitor, with their heads comfortably positioned in a chin rest. The screen was uniform grey with a mean luminance of 50 cd/m^2^. Superimposed on this background was the outline of a white circle 8° in diameter at the viewing distance of 94 cm (see Fig. 1). To test for voluntary generation of unpredictable visuo-motor behaviour, the participants were instructed to use the mouse to move the cursor as randomly and unpredictably as possible within the circle while clicking to record random cursor positions approximately twice a second. Each subject completed two runs comprising 625 cursor clicks each (total 1250). The x,y coordinates of each click and the temporal interval between successive clicks were recorded in order. As a control, each participant was subsequently asked to imagine a circle approximately half the diameter of the visible one and to make their clicks around that imaginary circle.

Experiments were repeated on three subjects using a circle of 12° diameter (2.25 times the area). No significant differences an any measurements were observed, thus serving as a control and indicating that the circle was sufficiently large to generalize to larger diameters. A further control that increased both circle diameter and click interval is discussed below.

Preliminary data analysis involved treating the x-coordinates as one time series and the y-coordinates as a second, with the results being averaged. However, statistically indistinguishable results were obtained by treating the x-coordinates followed by the y-coordinates as one time series of twice the length. Accordingly, the latter procedure was adopted throughout. All data analysis was conducted using Matlab™ programs written by the author.

Participants included 9 young (mean age 28.0, range 22–34 years) and 9 older adults (mean 67.2, range 56–81 years). Each age group contained 5 female and 4 male participants. As subsequent data analysis showed that there were no significant gender effects, gender is not discussed among the results.

Informed consent was obtained from all participants, and the experimental protocols were approved by the Office of Research Ethics at York University. In addition, all methods were performed in accordance with the relevant guidelines and regulations.

## Results

The goal of this study was to determine the extent to which participants could voluntarily generate a random or unpredictable sequence of clicks within the white circle outline on the screen. Complete data for one representative participant are illustrated in Fig. 1a,b, where each click under the unpredictable instruction is shown by a yellow cross in **a**, and each click under the imaginary circle instruction is illustrated in **b**. Typical temporal sequences of ten clicks during each trial are shown by a numbered sequence of red crosses in each panel. During the experiments participants only saw the current position of the cursor and the white circle, but no information regarding any past clicks. The data in Fig. 1a clearly exhibit a more complex structure than the roughly circular data cloud in Fig. 1b. Figure. 1c depicts the first 50 clicks in each 625 click sequence. There was no statistically significant correlation between these sequences, indicating that the two sequences were independent.

To determine whether the data were random, a standard runs test for randomness was employed^14^. This test first converts the time series data into a binary variable according to whether a value is greater than or less than the median of the series. The number of runs (R) is defined as the number of times there is a switch from above to below or vice versa. Calculation of a standard Z score estimates whether R is consistent with a random process. For the unpredictable condition Z = 13.29 ± 2.03 (sem). This value rejects randomness as an explanation for the unpredictable data at p < 10^−10^. This agrees with an earlier analysis of the literature that rejected randomness^6^. This leaves chaos or hyperchaos as the hypothesized mechanism.

**Figure 2:**
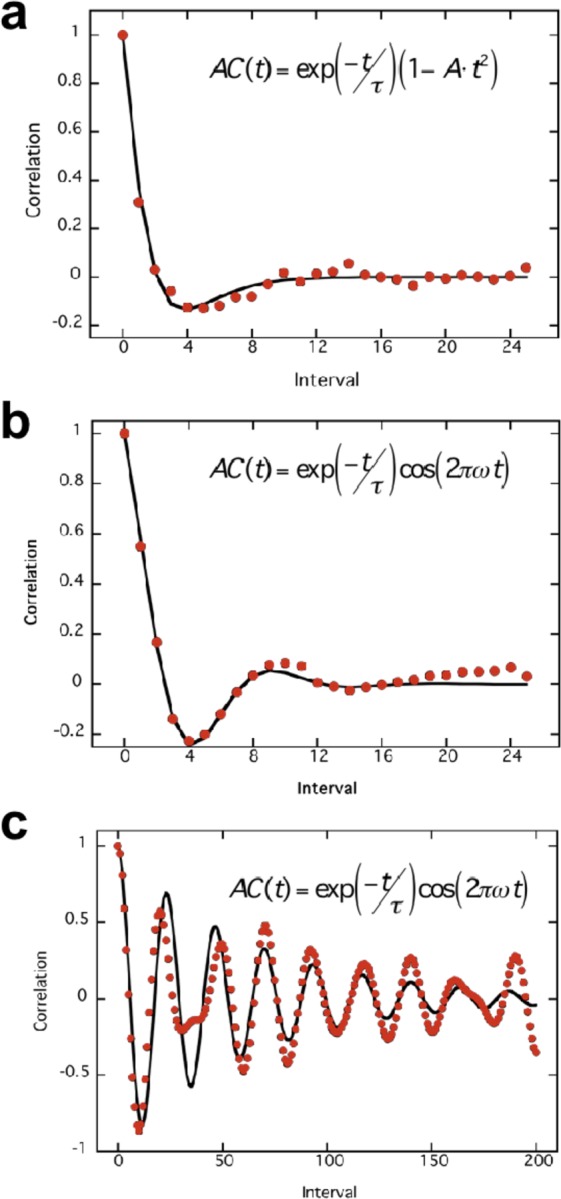
Click autocorrelation data as a function of offset in intervals and the best fitting equation under three conditions. (**a**) Autocorrelation function for one subject under unpredictable conditions that was best fit by Eq. (1). This equation provided the best fit for 10 subjects under unpredictable conditions. (**b**) Autocorrelation function best fit by Eq. (2), which provided the best fit to unpredictable data of 8 subjects. (**c**) Equation (2) provided the best fit to the data of all 18 subjects when clicking around an imaginary circle. Note that the abscissa extends to 200 intervals in this case, whereas it only extended to 25 intervals in (**a**,**b**). This reflects the very slow exponential decay of correlations in the circle condition relative to the rapid decay in the unpredictable condition. Panels (a,c) represent the subject’s data shown in Fig. 1a,b respectively.

One key signature of chaos is a rapid divergence of neighbouring trajectories, leading exponentially to unpredictability^15^. To quantify this, the temporal autocorrelation function of each click sequence was calculated^16^ and is plotted in Fig. 2a–c. The following two-parameter functions of time (t) were fit to each set of autocorrelation data:

1 $$AC(t)=\exp (-t/\tau )(1-A\cdot {t}^{2})$$

2 $$AC(t)=\exp (\,-\,t/\tau )\cos (2\pi \omega t)$$

where τ is the time constant (in click intervals) and A and ω are parameters representing fluctuations in the autocorrelation. AC(0) = 1 for both functions, as required for autocorrelation. Whichever function minimized the mean-square-error was chosen as the best fit. Equation (1) provided the better fit for the unpredictable data (Fig. 2a) for 10 of the 18 subjects, while the remaining 8 were better fit by Eq. (2) (Fig. 2b). Equation (2) provided the best fit to the circle data for every subject (Fig. 2c).

**Figure 3:**
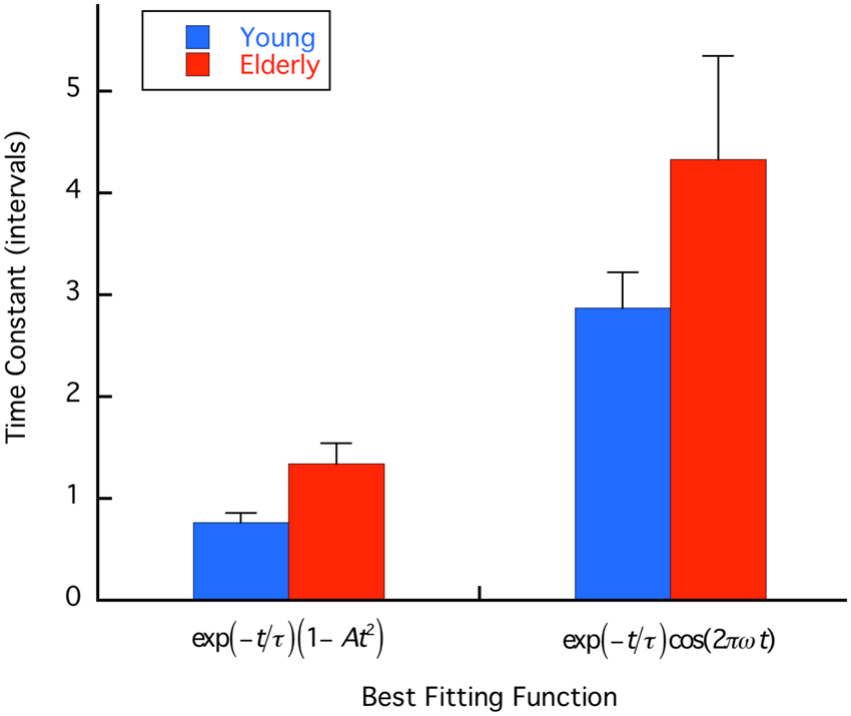
Time constants for autocorrelation exponential decay under unpredictable conditions separated by age group. Time constants were longer for Eq. (2) (right) than for Eq. (1) for both age groups. However, time constants for all elderly subjects (red) were longer than for their younger counterparts (blue) for each function. An ANOVA with age and function as the variables showed that this age difference was statistically significant. Error bars plot sem.

The exponential time constant τ for the unpredictable task is plotted for the two age groups in Fig. 3. A 2-way ANOVA was conducted on these data with the factors being age and best fitting function. Results showed that τ values were significantly different for the two function fits (F(1, 14) = 38.21, p < 0.0001). The effect of age was also significant (F(1,14) = 6.10, p < 0.028), and there was no interaction. As τ for the older group averaged 1.34 and 4.33 intervals for the first and second equations above, but 0.76 and 2.87 for the younger group, temporal autocorrelation decays more rapidly for the younger than the older group.

For the circular control task Eq. (2) provided the best fit for every subject, with τ = 22.11 and τ = 88.98 intervals for young and old respectively. This age difference was significant at p < 0.038. This again indicates a significantly faster autocorrelation decay for younger individuals. The decay constant τ for circles was also much greater for every subject than for the same subject under the unpredictable condition (p < 0.0005, paired t-test). Thus, all subjects retained information much longer in the circle condition.

On average, τ = 2.01 intervals for unpredictability and 55.44 for the circle control condition. As the mean inter-click interval was 0.43 ± 0.04 s with no significant difference between age groups or unpredictable versus circle conditions, the time constant for exponential decay was estimated as 0.86 s for the unpredictable condition and 23.84 s for the circle condition. Thus, predictability is rapidly lost in the former condition, as expected of chaos.

A second key test of hyperchaos involves computation of a fractal dimension such as the correlation dimension (D2)^17,18^. Calculation of D2 from a time series yields values close to 1.0 for an oscillation, values between 2.0–3.0 for simple chaos, and significantly higher values for hyperchaos. D2 for the circle condition averaged 1.77 ± 0.13 (sem), which is consistent with a limit cycle oscillation plus noise. For the unpredictable condition, however, D2 = 7.93 ± 0.05 (sem), which indicates hyperchaos of relatively high dimensionality (see below). This difference was statistically significant at p < 0.0001 (paired t-test).

As a third criterion, the 0–1 Chaos Test was also applied^19^. This test examines the temporal frequency spectrum of the time series through weighting the data by a large number of random frequency cosines and sines and averaging the amplitudes to produce a number K. As an oscillation has energy at only a small number of temporal frequencies, K ≈ 0. For chaos or hyperchaos, however, the continuous Fourier spectrum yields K ≈ 1. The average for our unpredictable condition was K = 0.92 ± 0.04, while for the circle condition K = 0.16 ± 0.03. This is indicative of chaos in the former condition and an oscillation in the latter. A paired t-test again showed this difference to be highly significant (p < 0.0001).

In a final control experiment, the circle diameter was increased to 12°, and the program was altered so that the recording clicks were required to occur at least 1.0 s apart. Four participants were tested on this procedure to determine whether the combination of an increased circle size and longer click interval might alter the results above. Subjects clicked at 1.56 ± 0.05 s, which averaged 3.7 times longer than in the original experiment. Most importantly, the statistical analysis of the results was unchanged: the runs test rejected randomness, and the remaining three tests supported hyperchaos. Thus, even the combination of slower recording over a larger circular region yielded the same results within the bounds explored here.

## Discussion

The results of this experiment show that humans can voluntarily generate unpredictable behaviour that is, in fact, hyperchaotic. However, in agreement with previous findings^6^, it is not random. These findings gain additional support from the physiological literature. There is evidence that primate cortex incorporates neurones that generate hyperchaos^20^. Similarly, the human EEG has been reported to be hyperchaotic^21–23^, and this is currently being explored by us on new data. On a behavioural level, there is also evidence that human perception and decisions might incorporate chaos^24–26^.

It should be noted that Ward^1,5^ has been justly critical of attempts to teach humans chaos using the logistic equation^7^. In fact, he emphasizes that introduction of imprecise (“fuzzy”) memory models can effectively mimic chaos. However, two aspects of the task in the present study indicate that this is not a problem here. First, no training or learning was involved. Second, the data are consistent with hyperchaos but not with simple chaos. Thus, the critique of the logistic equation, while appropriate, does not apply here.

Neural modelling has also indicated that cortical networks can generate chaos^27^. Work in my laboratory has shown that chains of coupled Wilson-Cowan neural oscillators^12^ can generate hyperchaos with D2 comparable to our experimental data. In particular, inhibitory to excitatory coupling of Wilson-Cowan neural oscillators has been shown to be efficacious in generating chaos^28^. Our results show that a model neural system with 6 positive Lyapunov exponents generates hyperchaos with a D2 comparable to current data (manuscript submitted).

Chaos and hyperchaos have been implicated in a number of neurological conditions. For example, autistic children are less responsive to viewing chaotic motion than are normal controls^29^. In addition, there is evidence that concussions reduce the level of chaos that is typical of motor control^30^. The effects documented above in normal aging raise the possibility that more dramatic reductions in the level of hyperchaos might be a marker for early stages of dementia. The simple chaos testing procedure described in this paper should prove useful in evaluating these possibilities.

Finally, this demonstration of voluntary hyperchaos generation is germane to the free will versus determinism controversy. Hyperchaos is a deterministic, dynamical mode of neural networks that clearly conform to the laws of physics. However, the decay of predictability in about 0.86 s indicates that neural hyperchaos is sufficiently unpredictable to fool both predators and other humans^8,9^. Thus, rather than “will” based on freedom from determinism, it may be more appropriate to think of hyperchaotic, unpredictable will, which is unpredictable even to the willing individual. Previous studies have shown a time lag in both EEG^31^ and fMRI^32^ signatures of decisions before subjects are consciously aware of their choices. These lags between neural decisions and consciousness of them would of necessity occur if these decisions were a result of hyperchaotic activity.
